# The Selah study protocol of three interventions to manage stress among clergy: a preference-based randomized waitlist control trial

**DOI:** 10.1186/s13063-021-05845-x

**Published:** 2021-12-09

**Authors:** Logan C. Tice, David E. Eagle, Joshua A. Rash, Jessie S. Larkins, Sofia M. Labrecque, Alyssa Platt, Jia Yao, Rae Jean Proeschold-Bell

**Affiliations:** 1 Duke Global Health Institute, Duke Center for Health Policy & Inequalities Research, Duke University, 310 Trent Drive, Durham, NC 27710 USA; 2 Department of Psychology, Memorial University of Newfoundland, St. John’s, Canada

**Keywords:** Stress, Intervention, Mindfulness, Patient preference, Controlled trial

## Abstract

**Introduction:**

Like many helping professionals in emotional labor occupations, clergy experience high rates of mental and physical comorbidities. Regular stress management practices may reduce stress-related symptoms and morbidity, but more research is needed into what practices can be reliably included in busy lifestyles and practiced at a high enough level to meaningfully reduce stress symptoms.

**Methods and analysis:**

The overall design is a preference-based randomized waitlist control trial. United Methodist clergy in North Carolina will be eligible to participate. The intervention and waitlist control groups will be recruited by email. The interventions offered are specifically targeted to clergy preference and include mindfulness-based stress reduction, Daily Examen, and stress inoculation training. Surveys will be conducted at 0, 12, and 24 weeks with heart rate data collected at 0 and 12 weeks. The primary outcomes for this study are self-reported symptoms of stress and heart rate at week 12 for each intervention compared to waitlist control; the secondary outcome is symptoms of anxiety comparing each intervention vs waitlist control.

**Ethics and dissemination:**

Ethical approval was obtained from the Duke University Campus IRB (2019-0238). The results will be made available to researchers, funders, and members of the clergy community.

**Strengths and limitations of this study:**

- While evidence-based stress reduction practices such as mindfulness-based stress reduction (MBSR) exist, a wider variety of practices should be tested to appeal to different individuals.
- Clergy in particular may prefer, and consequently enact, spiritual practices like the Daily Examen, and individuals such as clergy who spend most of their time thinking and feeling may prefer experiential-based practices like stress inoculation training.
- If efficacious, the Daily Examen and stress inoculation training practices have high feasibility in that they require few minutes per day.
- This study is limited by the inclusion of Christian clergy of only one denomination.

**Trial registration:**

ClinicalTrials.gov NCT04625777. November 12, 2020.

## Introduction

Members of helping professions, such as clergy, may be particularly susceptible to the adverse effects of chronic stress, particularly chronic work-related stress. Observational evidence reported by more than 1000 United Methodist clergy in North Carolina indicated that clergy exhibit high prevalence rates of chronic disease, including diabetes (12.7%), hypertension (36.2%), asthma (13.8%), and joint-related disease (33.8%), as well as obesity (41.2%) [1]. Further, in a sample of more than 1700 United Methodist clergy, clinically relevant symptoms of depression measured using the Patient Health Questionnaire-9 were reported by 11.1%, which is significantly higher than the adjusted national average of 5.5% [2]. The prevalence of clergy meeting the criteria for clinically relevant anxiety using the Hospital Anxiety and Depression Scale was also elevated at 13.5% [2]. The high prevalence of physical and mental health issues among clergy may be due in part to stressors from an emotionally demanding occupation with little respite.

One prominent theory of stress is the job-demand-control-support (JDCS) model [3], which indicates that stressful jobs are those characterized by high demand, low control, and low perceived support [4]. Clergy perform a wide variety of skilled roles, including inspiring the congregation, providing one-on-one care for congregants, performing sacraments, educating congregants, overseeing educational programming, and leading social justice activities. The work week typically averages 50 h or more with the expectation of being on call around-the-clock [5, 6]. While certain tasks such as preaching are predictable, clergy have no control over the timing of funerals and congregant crises, and only a variable degree of control over congregant perception and criticism of the direction that the clergy are taking the congregation. In terms of support, clergy experience work-related support to varying degrees; they direct a volunteer workforce and often do not receive the support needed to match the tasks or the emotional challenges faced [7].

Approaches to stress management can be categorized as *individual-level* or *organizational-level* depending on their target audience [8]. Numerous individual-level approaches have been developed to manage stress, such as cognitive-behavioral therapy, mindfulness, exercise, and relaxation. Meta-analyses report medium to large effect sizes for cognitive-behavioral approaches [9] and small to medium effect sizes for mindfulness- and relaxation-based approaches [9–11] to stress management. Learning to manage stress can be viewed as skill acquisition requiring engagement and practice. As such, choosing an intervention that individuals are willing and motivated to practice is a key consideration [12]. We sought to develop interventions that are tailored to the job demands and preferences of clergy in order to improve engagement and reduce the chronic negative impact of stress.

Our team conducted a pilot study with clergy to evaluate the feasibility and acceptability of four potentially stress-reducing interventions while taking participant preference into account. Three stress-reducing interventions showed promising trends in improving self-reported stress and/or physiological markers of parasympathetic nervous system activation. This manuscript reports the trial protocol for a waitlist-controlled preference design to evaluate the three interventions that were promising: mindfulness-based stress reduction, the Daily Examen, and a set of stress inoculation skills that we entitle stress proofing.

The goal of the Selah study is to test the efficacy of each of three flexible and scalable stress management interventions among clergy who serve in a high-stress occupation. The approaches of the three interventions differ; by determining whether each intervention is superior to no treatment on stress symptoms, anxiety symptoms, and heart rate variability, study findings will indicate evidence-based program options for clergy, based on their preferences.

## Methods

### Research objectives

#### Objective 1

To compare the impact of, separately, MBSR, the Daily Examen, and stress proofing with a waitlist control condition, on stress outcomes at 12 weeks.

Hypotheses 1 and 2: When randomly assigned to waitlist vs non-waitlist, MBSR, the Daily Examen, and/or stress proofing will be superior to the waitlist control on stress symptoms (hypothesis 1) and HRV (hypothesis 2) at 12 weeks (co-primary outcomes).

#### Objective 2

To compare the impact of, separately, MBSR, the Daily Examen, and stress proofing with a waitlist control condition, on anxiety symptoms at 12 weeks.

Hypothesis 3: When randomly assigned to waitlist vs non-waitlist, MBSR, the Daily Examen, and/or stress proofing will be superior to the waitlist control on anxiety symptoms at 12 weeks (secondary outcome).

#### Objective 3

To compare the impact of, separately, MBSR, the Daily Examen, and stress proofing with a waitlist control condition, on stress and anxiety symptoms at 24 weeks.

Hypotheses 4 and 5: When randomly assigned to waitlist vs non-waitlist, MBSR, the Daily Examen, and/or stress proofing will be superior to the waitlist control on stress symptoms (hypothesis 4) and anxiety symptoms (hypothesis 5) at 24 weeks (exploratory outcomes).

#### Objective 4

To determine whether having a preference, and that preference being honored, was associated with better outcomes on stress symptoms, HRV, and anxiety symptoms.

Hypotheses 6–8: Participants who had a stated preference and received that intervention (i.e., MBSR, the Daily Examen, and stress proofing combined) will experience larger between-arm (waitlist vs non-waitlist) differences in improvements on stress symptoms (hypothesis 6), HRV (hypothesis 7), and anxiety symptoms (hypothesis 8) at 12 weeks when compared to no-preference participants randomly assigned across interventions and waitlist (exploratory outcomes).

### Study design: partially randomized waitlist-controlled preference trial

**Fig. 1:**
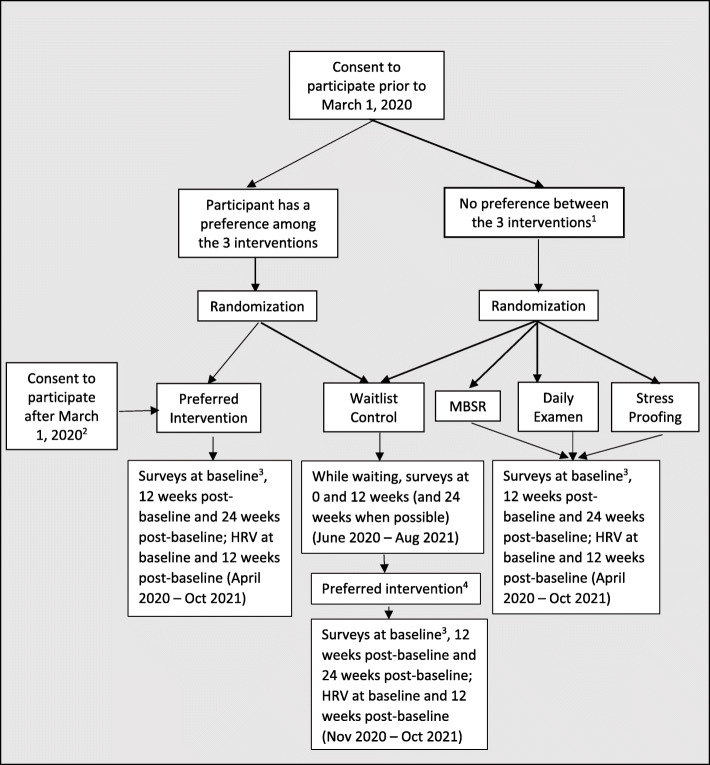
Pandemic-adapted Selah study design: a partially randomized waitlist-controlled preference

This study’s timing crossed the start of the COVID-19 pandemic, which necessitated changes in the original design registered with clinicaltrials.gov (see Fig. 1). In this manuscript, we report the pandemic-adapted design (see Fig. 1), a partially randomized waitlist-controlled preference trial with assignment into non-waitlist (i.e., intervention) vs waitlist arms. Preference-based trials are a kind of pragmatic clinical trial design that recognizes individuals have treatment preferences that are likely to affect the outcomes and result in “preference effects” [12]. These preference effects may be due to expectancy effects or degree of engagement, which are particularly important in behavioral interventions. In conventional randomized controlled trials, participant choice is removed to create high internal validity between the intervention and treatment effects [13]. Blinding participants to the allocated intervention can be challenging in behavioral trials, making participant preferences more salient.

We enacted a partially randomized preference design to assign participants to particular interventions [12]. Prior to March 1, 2020, and the COVID-19 pandemic, participants read the descriptions of the three stress management interventions and provided preference ratings using the Treatment Acceptability and Preferences Scale [14] to help them think through their preferences. In addition, participants were asked whether they preferred any of the three interventions or if the interventions were equally appealing to them (i.e., no preference). Those with a preference were asked whether they had one or two equal first choices. Participants who preferred one intervention were assigned to their preferred intervention and randomized to a non-waitlist vs waitlist condition; the waitlist condition participants provide control data prior to intervention receipt. Upon completing the waitlist condition, participants were allowed to update their preference and receive their currently preferred intervention while providing data. Participants who stated no preference (or equally preferred two interventions over the third) were randomized to receive one of three (or, if applicable, two) interventions in a non-waitlist condition or to the waitlist condition (Fig. 1).

During a time of heightened stress due to COVID-19, stress reduction research seemed particularly important, and we sought more participants to maximize statistical power should others withdraw [15]. For those reasons, after March 1, 2020, we continued to recruit participants and assigned them to their preferred intervention in a non-waitlist condition (Fig. 1). These additional recruits will provide observational, as-treated data.

### Study setting

Clergy from the United Methodist Church (UMC) appointed to positions in North Carolina (NC) will be recruited. There are approximately 1600 active UMC clergy in this potential study group. The majority serve as congregational leaders, but some work in non-profit and denominational settings in rural, suburban, and urban environments. The average age is 53, 66% are men, and 90% are white and non-Hispanic [16].

### Patient and public involvement

The material introduced in the interventions was pilot-tested by 78 clergy prior to the final development of the intervention protocols.

### Participant eligibility

#### Inclusion criteria

Participants must have had a current appointment in the 2019–2020 or 2020–2021 appointment cycle of the NC Annual Conference or the Western NC Annual Conference of the UMC to be included in the study. Participants are eligible if they are 18 years of age or older and willing to participate in the survey and HRV data collection and commit to completing their assigned stress management intervention. There are no stress- or health-related inclusion criteria.

#### Exclusion criteria

In an attempt to increase ecological validity, no exclusion criteria were set for participation in the trial. Participants with underlying medical conditions which could seriously impact the integrity of their HRV data were excluded from HRV data collection, including a diagnosis of tachycardia; being pregnant or becoming pregnant during the course of data collection; being diagnosed with COVID-19; having a pacemaker; and documentation of other cardiovascular-related chronic or acute morbidities.

### Procedures

#### Screening, recruitment, and enrollment

Recruitment is expected to benefit from the 12-year partnership that the Duke Clergy Health Initiative and the two UMC conferences in NC have enjoyed. Participants will be recruited primarily via email addresses provided by the denomination. Additionally, all eligible clergy will receive a program brochure in the mail. Study staff, including a recruitment coordinator who is well-known to the population, will promote the study and answer questions at in-person gatherings. Participants will be directed to the study website (spiritedlife.org) to learn more about the interventions and study details. The web-based registration form will guide participants through a series of questions that will culminate in indicating their preferred intervention(s) and study consent. Participants will be compensated $20 for each of the first two surveys and $25 for the final survey, as well as $25 for each occasion of heart rate tracking submitted (possible total = $115).

#### Allocation concealment mechanism

In the current study, the consideration for allocation concealment was concealing assignment to waitlist vs non-waitlist. Participants enrolled prior to assignment; therefore, knowledge of waitlist vs non-waitlist allocation by study staff or participants was not possible at the time of enrollment.

#### Randomization and blinding

For participants recruited before March 1, 2020, after enrollment, participants were randomly allocated to waitlist vs non-waitlist using computer assignment. The analysis statistician wrote the randomization codes in Stata version 16. The allocation sequence generation used stratified randomization, stratifying based on intervention preference (see Table 1). Random assignment to an intervention type was performed using a random draw from a uniform distribution. Random assignment to immediate vs waitlist condition was performed by drawing a random sample from a list of participants within each intervention type assignment. To keep the analysis statistician blinded, a research staff member ran the Stata program and gave the assignment information to trial coordinators who then coordinated logistics. Trial coordinators had no prior knowledge of potential assignment to waitlist vs non-waitlist at the time of recruitment or enrollment. Table 1 reports assignment and randomization allocations for each preference scenario. Preference data were collected before allocation, and baseline data (e.g., on stress and anxiety symptoms) were collected after allocation. A secondary recruitment period was initiated after March 1, 2020, that did not include random assignment; data from those participants will be included in a secondary as-treated analysis. The analysis statistician will be blinded to the intervention allocation. It is not possible to blind participants, interventionists, or outcome assessors.

**Table 1:** Study assignment approach for each preference and enrollment date scenario

Preference scenario	Assignment approach
**Enrolled prior to March 1, 2020**	
No preference between interventions	Randomly assigned to one of the four study arms: three interventions without waitlist and the waitlist arm, with a 1:1:1:1 ratio.
Preferred two interventions equally and over the third intervention	Randomly assigned to one of the two interventions with a 1:1 ratio, and then, a fraction was randomly assigned to the waitlist arm, with a 3:1 non-waitlist vs waitlist ratio for MBSR and stress proofing and a 5:4 non-waitlist vs waitlist ratio for the Daily Examen (DE was preferred by more participants and this allowed the same number of participants to be randomized into each study arm).
Preferred one intervention among the three	Assigned to their preferred intervention and combined with participants with two top preferences who had been randomized to that intervention, and then randomly assigned to non-waitlist vs waitlist arms, with a 3:1 non-waitlist vs waitlist ratio for MBSR and stress proofing and a 5:4 non-waitlist vs waitlist ratio for the Daily Examen.
Any of the above scenarios and are part of a married or cohabitating couple who both meet study criteria and enrolled	To avoid spillover effects, each couple was treated as if they were one person, i.e., assigning both spouses to the same intervention and randomizing them into a non-waitlist vs waitlist arm. When a couple had different preferences, one preference was randomly chosen as the couple’s preference.
Seven clergy with an established meeting group (a covenant group)	The seven clergy jointly chose a single preferred intervention and were randomized together to the non-waitlist vs waitlist arm.
**Enrolled after March 1, 2020**	
All enrollees after March 1, 2020, with no preference, two equal preferences, or one preference, and whether a clergy couple or not	Participants answered treatment preference survey items, but regardless of their answers, self-selected the intervention with intervention dates they most wanted from the full list of workshop options. None was randomized to the non-waitlist vs waitlist arms; they all were assigned to non-waitlist.

#### Study arms: non-waitlist interventions and waitlist

This trial includes three non-waitlist study arms (mindfulness-based stress reduction, Daily Examen, and stress proofing) and one waitlist arm that includes two conditions: first providing control data and then participating in any intervention condition. Originally, participants were to be trained in small workshop formats delivered across NC between April 2020 and May 2021. Due to the global pandemic, all workshops were converted to online delivery formats and delivered during the same anticipated months. Interventions will be delivered in small groups of 10–25 participants. Survey data will be collected online, and HRV data will be collected by the participants in their respective personal settings.

##### Mindfulness-based stress reduction (MBSR)

MBSR teaches several different kinds of meditation and attitudes. The specific course for this study is a synchronous web-based video platform course with certified instructors from Duke Integrative Medicine and content based on Jon Kabat-Zinn’s model [16, 17]. It includes exercises in awareness of breath, body scans, walking meditation, “choiceless” open awareness, loving kindness meditation, and bringing awareness to the present moment. The course consists of 8 weekly sessions confined to study participants held via video conference with meditation instruction, periods of guided practice, and group discussion. Participants are also offered a 4-h online “day of mindfulness” which includes both participants and community members not enrolled in the study.

##### The Daily Examen (DE) prayer practice

The Daily Examen is a Jesuit reflective prayer practice that was first developed by Ignatius of Loyola and widely practiced by Christians from many traditions. We used a modern adaptation of the Daily Examen [18].

The Daily Examen guides the person through a five-step prayer:

1. Become aware of God’s presence.
2. Review the events of the past 24 h, recalling 2–3 things for which you are grateful.
3. Review the events of the past 24 h, guided by the Holy Spirit, noticing where you experienced God’s presence.
4. Review what stands out and pay attention to what emotions arise. With the guidance of the Holy Spirit, pray through these emotions, noticing which are drawing you closer to God or pulling you away from God.
5. Look forward to the next 24 h. What is the one thing you should do? Where do you need God’s assistance?

The Daily Examen is designed to help the supplicant reflect on positive emotions, move past negative emotions, and align their work with God’s work. Instructors trained in Ignatian spirituality and regular practitioners of the Daily Examen developed workshop content that included three occasions of practicing the Daily Examen, lecture instruction, and small group discussion. Topics covered include a history of Ignatian spirituality, the emotions and the spiritual life, and the practicalities of developing a daily prayer practice. We asked the participants to conduct the Daily Examen as a daily practice, requiring a 10–15-min commitment for 6 months following their workshop. Participants were also offered the opportunity to meet with their instructors in an online small group format 2 and also 6 weeks following their workshop to address any issues arising from their practice.

##### Stress proofing (SP) inoculation combination

Stress proofing is a set of stress reduction skills with aspects of stress inoculation training [19], selected and organized by the founder of an organization called NC Systema. The founder designed and led a weekly, synchronous, web-based workshop for 4 weeks. Consistent with stress inoculation training, the workshop began with education on the stress response and physical awareness of one’s own stress response [19, 20]. The training diverges from traditional stress inoculation training and goes on to focus on physical activities to undo the stress response. These activities include walking with diaphragmatic breathing, triangle and rectangle breathing, tension control, stretching, and massage. Stress inoculation training is discussed, and participants are encouraged during periods of less stress to allow themselves a degree of physical discomfort to learn to tolerate discomfort in the future [21]. The training recommends a variety of beneficial lifestyle practices, including prioritizing nutrition and sleep and disengaging from technology for several hours before sleep. The daily practice plan emphasized stress awareness and diaphragmatic breathing, with encouragement to try the lifestyle adjustments for a week at a time.

##### Waitlist condition

Participants allocated to the waitlist arm will undergo a 24-week waiting period with the purpose of providing control data, during which they will respond to surveys (0, 12, and 24 weeks). Those who are eligible will also provide a 48-h continuous ambulatory heart rate recording at 0 and 12 weeks. Upon completing the waiting period, participants will be informed that they can update their intervention preference and receive that preferred intervention while providing intervention data.

We recognize that a waitlist control condition sets a “low bar” for evaluating efficacy among interventions [22]. We decided not to use an active control condition because two of our three stress management interventions have not been evaluated as a strategy to improve stress symptoms relative to a no-treatment control group, the recommended first step in intervention evaluation. As such, it is premature to invest considerable resources and compare the Daily Examen and stress proofing to an active control before demonstrating efficacy over a waitlist control group.

#### Qualification of interventionists

Instructors for each of the three interventions were hired based on their certifications and knowledge of the specific content area. Mindfulness-based stress reduction instructors are certified instructors who have been vetted and hired by Duke Integrative Medicine’s MBSR program. Stress proofing is delivered by the founder of a North Carolina-based health coaching business. While the program has been offered to a variety of local employers, significant changes were made to it to adapt it to a clergy population. The Daily Examen instructors are certified spiritual directors trained in Ignatian spirituality with experience working with clergy and religious professionals.

### Study measures (see Table 2)

#### Primary outcomes

The *Calgary-Symptoms of Stress Inventory* (C-SOSI) is a 56-item, 8-subscale measure of self-reported stress symptoms [23]. We included the subscales of anger (8 items), muscle tension (8 items), cardiopulmonary arousal (6 items), neurological/gastroenterological (10 items), and cognitive disorganization (9 items) that total 41 items. Validation studies have shown convergent validity with specific subscales and overall divergent validity with anxiety [23]. Response options for each item range 0–4. We will use continuous mean scores of all subscales combined; higher mean scores indicate worse symptoms.

**Table 2:** Measures by study objective and time point

Measure	Objective^a^	At consent	0 weeks^b^	12 weeks	24 weeks
**Primary outcomes**					
Calgary Symptoms of Stress Inventory (adapted)	1, 3, 4		X	X	X
48-h ambulatory heart rate	1, 4		X	X	
**Secondary outcome**					
General Anxiety Disorder-7	2, 3, 4		X	X	X
**Other pertinent measures**					
Gender, age, race, Hispanic ethnicity, bi-vocational status	Covariates for primary, secondary, and exploratory analyses		X	X	X
Godin-Shephard Leisure-Time Physical Activity Questionnaire, body mass index, caffeine intake, alcohol consumption	Additional covariates for HRV analyses		X	X	X
Preference for online vs in-person interventions, marital status, children living at home, clergy appointment	Covariates for sensitivity analyses		X		
Financial stress, Patient Health Questionnaire-8 (depressive symptoms), marital status, number of hours worked per week as a clergy, morale of congregants	Covariates for sensitivity analyses		X		
Treatment Acceptability and Preferences Scale	Inform their preference decision; covariate for sensitivity analyses	X			

^a^See the “Research objectives” section in the manuscript

^b^Zero weeks for controls; immediately pre-intervention for the intervention group

#### Heart rate

A subset of participants will be provided with an ambulatory heart rate monitoring device to wear for a 48-h period during week 0 and week 12, during which time participants proceeded with their usual daily routines, including exercising, bathing, and sleep activities.

Heart rate will be measured using continuous electrocardiographic (ECG) recording sampled at a rate of 1000 Hz and used to calculate *heart rate variability*. Participants will be fitted with an eMotion Faros 180 ambulatory heart rate recording device (Bittium) connected by electrode leads to two pre-gelled (Ag/AgCl) disposable Ambu BlueSensor wet-gel ECG electrodes attached beneath the right clavicle and left ribcage. The 48-h ECG recording will be imported to the Kubios HRV Premium V3.4.1 software [24], partitioned into 5-min segments, visually inspected to allow for manual correction of ectopic beats, detrended, and subject to Kubios’ automatic artifact correction algorithm [25]. Heart rate variability will be indexed using the time-domain metric *root mean square of successive RR differences* (*RMSSD*) given that it is less affected by breathing and a better suitable outcome measure in ambulatory studies than frequency-domain measures [26].

Following recommendations for the detection of circadian rhythmicity [27], 5-min segments across 24 h of recording will be subject to a cosinor analysis using the Cosinor package for R. Two individual-level cosine function parameters will be estimated by linear models with ordinary least square estimations to quantify the circadian variability parameters: (i) midline estimating statistic of rhythm (MESOR), defined as the rhythm adjusted 24-h mean, and (ii) amplitude, defined as the distance between MESOR and the maximum of the cosine curve (i.e., half the extent of rhythmic change in a cycle).

#### Secondary outcome

Symptoms of anxiety will be measured using the *Generalized Anxiety Disorder-7* (GAD-7) [28], a self-report measure of the extent to which respondents have been bothered by 7 symptoms of generalized anxiety, corresponding to the DSM-IV criteria, over the past 2 weeks using a 4-point Likert scale from 0 “not at all” to 3 “nearly every day.” The sum scores range from 0 to 21 with scores of ≥ 5, ≥ 10, and ≥ 15, representing mild, moderate, and severe levels of anxiety, respectively. Psychometric properties of the GAD-7 are well documented [29].

#### Pertinent demographics measures

A demographics questionnaire was designed to measure *sex* (male/female), *age* (in years), *race*, *ethnicity*, self-reported *physical and mental health conditions*, *marital status*, and having *children living at home*. To capture work-related characteristics that may relate to stress, we will measure *bi-vocational status* (i.e., job in addition to serving as clergy) and *clergy appointment*, i.e., whether they serve in a church or not.

#### Additional measures

Physical activity levels will be measured using the *Godin-Shephard Leisure-Time Physical Activity Questionnaire* [30], a self-report measure of how often in the past 7 days and for how many minutes per time one has engaged in physical activity, measured separately for strenuous, moderate, and mild exercise. We will use self-reported weight and height to assess *body mass index* [31]. We will use single items to assess average daily *caffeine intake*, average weekly *alcohol consumption*, *financial stress* (how stressful is your current financial situation for you? not at all-extremely), *number of hours worked* per week, and *preference for online vs in-person intervention*. *Depressive symptoms* will be measured using the self-report eight-item Patient Health Questionnaire (PHQ-8) which asks about the frequency of specific depressive symptoms experienced within the past two weeks [32, 33].

### Timeline of assessments

#### Surveys

**Fig. 2:**
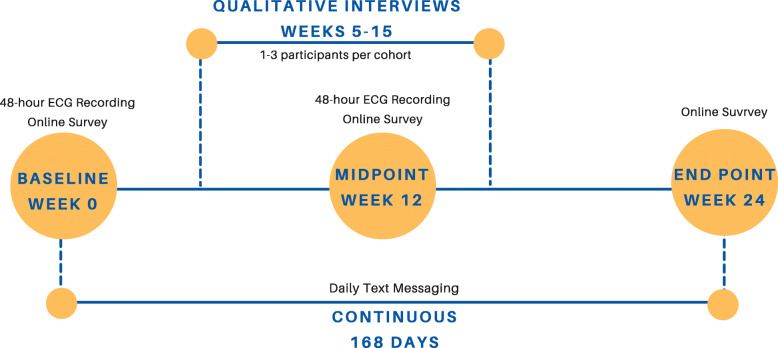
Timeline of measures: surveys, ECG, and daily practice times (reported via text messaging)

Participants will complete the study measures immediately before starting the intervention (baseline), 12 weeks after commencing the intervention, and 24 weeks after commencing the intervention (see Fig. 2). Participants will be prompted to complete the surveys via email and given a link to an online survey whose data are securely stored in REDCap. The data collection window will be from 1 week prior to 3 weeks after the time point.

#### Texting

Participants will receive daily text messages at noon that inquire their practice from the previous day. In the case of stress proofing, the text asks how many daily resets were performed; for MBSR, the number of minutes of formal meditation practiced; and for the Daily Examen, whether they have prayed the Examen prayer or not. Participants who have not responded by 4 p.m. will receive a reminder. Text messages will be distributed through an automated system generated by programmers at Duke Digital Health.

#### Qualitative interviews

Purposive samples of participants from each intervention group will be invited to participate in in-depth interviews about their experiences. Interviews will be performed between 5 and 15 weeks after starting the intervention with the goal of evaluating acceptability, perceived value, benefits, unintended consequences, and intervention delivery. We will select up to three participants in early workshop groups as we refine the intervention delivery and 1–2 participants in later workshop groups. Participants will be selected using their text message engagement data, to include both high- and low-practice participants. Interviews will be conducted using semi-structured interview guides and audio-recorded. The interviewer will take comprehensive notes and complete a post-interview debrief form. Interview notes will be coded in qualitative software using a pre-defined book of index codes [34]. Content analysis will be conducted to achieve the goals stated above.

### Participant engagement

Participant engagement will be encouraged through multiple methods. Participants will acknowledge their understanding of the three core aspects of participation (workshop attendance, surveys, and heart rate tracking), and commit to participate in all aspects. Second, participants will be oriented to the study as they prepare to begin their intervention (or ask questions via phone, email, or Zoom for controls). At this time, participants are given program materials and study-branded items such as coffee mugs, water bottles, and pens, plus information about the compensation for completing each of three surveys and two occasions of heart rate tracking. Course instructors and the research team will contact participants via email to remind them of upcoming class sessions and surveys. Participants will have the opportunity to engage further with their instructors in online follow-up sessions (2 weeks and 6 weeks for DE; 1 month for SP). The daily text message to collect the amount of practice also serves as a daily engagement prompt. Lastly, the participants will receive “thank you” correspondence in the mail prior to being invited to complete their final survey.

### Analysis

#### Statistical analysis plan

##### Power analysis

A baseline average C-SOSI score of 0.92 (SD = 0.46) and average 12-week follow-up scores of 0.7 (SD = 0.58) for MBSR, 0.55 (SD = 0.36) for stress proofing, and 0.51 (SD = 0.38) for Daily Examen are expected based on study pilot data. Given an alpha of 0.0167 (based on a Bonferroni correction to hypothesis tests for the effects of 3 interventions on C-SOSI scores), a per-arm sample size of 195 for MBSR, 40 for Daily Examen, and 47 for stress proofing will yield 80% power to detect a between-arm difference in the means at 12 weeks of 0.22 for MBSR, 0.37 for stress proofing, and 0.41 for Daily Examen for a two-sample *t*-test with unequal variances, allowing for a loss-to-follow-up of 20% and a design effect of 1.3 (corresponding to an ICC of 0.027 and average cluster size of 12) to account for clustering caused by the group-based intervention delivery.

Previous literature recommends that medium effect size (difference in means/standard deviation) for HRV be defined as 0.50. A per-arm sample size of 140 will yield 80% power to detect an effect size of 0.50 for a two-sample *t*-test with an alpha of 0.0167, allowing for a loss-to-follow-up of 20% and a design effect of 1.3 to account for the group-based intervention delivery. All power analyses were performed using the PASS 2021 software.

##### Data analysis

All primary and secondary study outcomes are measured on continuous scales and will be reported with mean estimates and 95% confidence intervals using the intention-to-treat principle with reference to the waitlist vs non-waitlist for participants randomly assigned prior to March 1, 2020. Linear mixed models will be specified with fixed effects for time and intervention status, and partially clustered random effects for the non-waitlist arms will be used to account for the clustering caused by workshop-level intervention delivery that affects participants in the intervention condition vs the un-clustered structure for those in the waitlist condition. Distributions of residuals will be examined to confirm that modeling assumptions are met. Models with adjusted and unadjusted estimates will be specified, with adjustment covariates such as age, sex, and bi-vocational status chosen prior to unblinding and finalization of the analysis plan and sensitivity analyses to include any additional relevant covariates that are identified. We will include race and ethnicity contingent upon there being meaningful heterogeneity. We may choose to adjust for baseline stress level to improve the precision of the treatment effect. In addition, for the models evaluating HRV, an a priori decision was made to include baseline sex, age, caffeine intake, alcohol consumption, body mass index, and physical activity as covariates given the influence that these variables exert on HRV [35]. For the primary outcome of stress symptoms, *p*-values will be calculated for the 12-week differences for each of the 3 interventions vs waitlist and will be adjusted for multiple comparisons using the Benjamini-Hochberg correction [36].

The effects of preferences on intervention estimates will be ascertained by introducing binary indicators for receipt of intervention preference vs assignment by random choice, which will be interacted with intervention indicators. Because this study is not powered to detect such interaction effects, the analytic focus will be on the magnitude of interaction effects and their 95% confidence intervals.

We will perform sensitivity analyses adjusting for any influential covariates (e.g., baseline financial stress, depressive symptoms, marital status; see Table 2) that are imbalanced by chance between waitlist and non-waitlist arms.

Due to the effects of the COVID-19 pandemic on recruitment, intervention delivery, and potential follow-up rates, analyses will be approached from two angles. First, analyses will be performed using data from participants recruited prior to March 1, 2020, when the randomized-waitlist design was applied. Standard analytic approaches applicable to randomized studies and CONSORT reporting will be applied. Second, participants recruited prior to March 1, 2020, will be pooled with those recruited after March 1, 2020, and data will be analyzed with methods appropriate to observational study designs using as-treated methods (e.g., propensity score weighting to account for treatment selection).

If 5% or more of participants miss follow-up data, a sensitivity analysis will be performed using propensity score or multiple imputation methods to account for potentially informative missingness, and estimates will be compared to the original complete case estimates.

##### Data monitoring, risk management strategies, and auditing

The study does not have a data monitoring committee because of minimal anticipated risks from the interventions. We did not anticipate a need to stop the trial early for safety or efficacy reasons, and thus an interim analysis was deemed unnecessary. Adverse outcomes will be monitored through participant and instructor reporting to the research team and through in-depth interviews. The study does not include planned auditing.

##### Ancillary and post-trial care

The anticipated study risks are minimal, and ancillary or post-trial care will not be offered. The study team will not discourage participants from independently augmenting their assigned intervention with other stress reduction methods (e.g., exercise, professional coaching) to assist with their stress. The interventions were designed to be applicable and sustainable long after the trial ended.

### Ethics and dissemination

All procedures of protocol 2019-0238 were approved by the Duke University Campus Institutional Review Board (IRB). Changes to the protocol that affect the study aims, research design, sample size calculation, participant safety, participant benefit, or study procedures will result in a formal amendment to the IRB protocol and require approval from the IRB prior to implementation. The results from this trial will be disseminated to the academic community through peer review manuscripts; results will be posted to the Clergy Health Initiative website and made available to participants, denominational clergy leadership, and the general public.

#### Data management

Data will be collected, de-identified, and stored securely on Box and REDCap hosted by Duke University. De-identified data will be made available on reasonable request where such requests are compliant with receipt of ethical approval from the sending and receiving the hosts’ institutional ethics review boards.

### Implications

The goal of the Selah study is to provide one or more replicable stress management programs with demonstratable effectiveness for improving stress management skills among clergy. The study is designed to find a practice that is flexible and capitalizes on participant preferences. To this end, we have included a “gold standard” stress management intervention, a cognitive-behavioral intervention, and a promising spiritual practice. The cumulative pattern of the results will inform which intervention will be likely to have the best benefit for a particular individual given their current circumstances and preferences. We will translate the observed results into actionable recommendations provided to clergy stakeholders. The purpose of the recommendations will be to facilitate stress management skills and reduce symptoms of stress among clergy.

### Trial status

The Selah Trial ClinicalTrials.gov number is NCT04625777, and the Duke University Campus Institutional Review Board number is 2019-0238. Recruitment began on January 6, 2020, and the end of data collection was on September 24, 2021.

## Data Availability

Please contact the authors for data requests.
